# Mean Platelet Volume as a Predictor of COVID-19 Severity: A Prospective Cohort Study in the Highlands of Peru

**DOI:** 10.3390/diseases10020022

**Published:** 2022-04-15

**Authors:** Jhosef Franck Quispe-Pari, Jose Armando Gonzales-Zamora, Judith Munive-Dionisio, Cristhian Castro-Contreras, Abelardo Villar-Astete, Cesar Kong-Paravicino, Pierina Vilcapoma-Balbin, Jorge Hurtado-Alegre

**Affiliations:** 1 Infectology Unit, Department of Medicine, Hospital Nacional Ramiro Prialé Prialé, Huancayo 12004, Peru; jhosefqp@gmail.com (J.F.Q.-P.); junicolemunive@yahoo.es (J.M.-D.); pierina222@hotmail.com (P.V.-B.); jorge_13_1991@hotmail.com (J.H.-A.); 2 Faculty of Human Medicine, Universidad Nacional del Centro del Peru, Huancayo 12004, Peru; 3 Infectious Disease Division, Department of Medicine, Miller School of Medicine, University of Miami, Miami, FL 33136, USA; 4 Peruvian American Medical Society, Albuquerque, NM 87111, USA; 5 Clinical Pathology Unit, Department of Diagnostic, Hospital Nacional Ramiro Prialé Prialé, Huancayo 12004, Peru; sale53722cf@gmail.com (C.C.-C.); cesar.kongp@gmail.com (C.K.-P.); 6 Radiology Unit, Department of Diagnostic, Hospital Nacional Ramiro Prialé Prialé, Huancayo 12004, Peru; abe76s@hotmail.com; 7 Facultad de Medicina Humana, Universidad Continental, Huancayo 12004, Peru

**Keywords:** COVID-19, mean platelet volume, altitude, severity, predictor, meters above sea level

## Abstract

Introduction: Although 80% of symptomatic individuals with COVID-19 develop mild forms, it is the severe (15%) and critical (5%) forms that have the greatest impact in the hospital setting. Recognizing markers that can predict severe forms is essential, especially in high-altitude populations. Methods: We conducted a prospective cohort study at 3200 masl (meters above sea level) in a city in Peru to determine if MPV (mean platelet volume) level is a predictor of COVID-19 severity. Patients with mild/moderate disease were enrolled and followed for 21 days or until the development of severe disease (primary outcome). A bivariate analysis was used to identify variables associated with severe disease. A ROC analysis determined the best MPV (mean platelet count) cut-off to predict COVID-19 severity, and then, a multiple regression analysis was performed. Results: 64 patients were enrolled. The median age was 48.5 years (IQT 39–64.5) and the proportion of women was 51.6%, the most frequent symptoms were chest pain (73%), fever (71%), and dyspnea (67%). The median time to develop a severe form from the onset of symptoms was 11 days (IQT 10.5–13). The most common radiographic phase on CT scan (computed tomography) was progressive (60.38%). We observed that an MPV of more than 10.15 fL in the first week of disease predicted severity regardless of age and sex at high altitudes. Conclusions: MPV in the first week of the disease may predict severity in patients diagnosed with COVID-19 at high altitudes; however, we need prospective studies with a larger population and at a different altitude, levels to confirm these findings.

## 1. Introduction

Since the appearance of the first cases of severe acute respiratory syndrome coronavirus 2 (SARS-CoV-2), the scientific community has been trying to resolve doubts about this terrible pathogen and its implications for human health. Although 80% of symptomatic infected patients develop mild forms, it is very important to determine who would develop severe (15%) and critical (5%) disease, because it has enormous implications for the healthcare system, particularly in hospital bed occupancy and availability of resources [1]. To date, several demographics, clinical, and laboratory markers have been recognized as associated with the severity and mortality of COVID-19 [2,3]. For instance, it has been described that MPV is higher in patients with COVID-19 when compared to those without SARS-CoV-2 infection [4,5,6].

The mean platelet volume (MPV) is the size of platelets measured through automated blood biometry [7], which normal value is 8.81 +/− 1.68 fL [8,9]. This marker reflects the presence of young platelets in the blood, probably due to megakaryocyte hyperproliferation in the bone marrow [10]. Larger platelets are functionally, metabolically, and enzymatically more active than smaller ones. They contain more intracellular thromboxane A2 and increased expression of procoagulant surface proteins such as p-selectin and glycoprotein IIIa, causing greater prothrombotic potential [11]. Although MPV is not frequently used in medical practice, its usefulness has been evaluated in thrombocytopenia, in addition, to being a risk/prognostic marker for cardiovascular, thrombotic, and inflammatory diseases, as well as sepsis [12,13]. As a predictor of COVID-19 severity, an elevated or rising MPV has been also associated with mortality; however, there is no conclusive data [14].

Regarding the high-altitude population and COVID-19, the information is very limited. There is literature that suggests that altitude plays a protective role in COVID-19 incidence and severity [15,16]. In contrast, other studies have found that altitude is associated with increased mortality [17,18,19].

Due to the variability of these findings and the scarce information on high-altitude populations, we conducted a study in Peru at 3200 masl to determine whether MPV level is a predictor of COVID-19 severity.

## 2. Methods

### 2.1. Study Design

An observational, analytical, prospective cohort study was conducted on patients with COVID-19. The infection was defined by a positive IgG/IgM serologic test, nasopharyngeal PCR, or nasal antigen test, plus compatible pulmonary computed tomography (CORADS 4 or 5) [20].

Informed consent was obtained prior to the enrollment of participants. This study was approved by the EsSalud ethics committee on 23 June 2020, and it was registered in the Peruvian National Institute of Health with the code 2BE654C8-98D7-47C6-B27F-0DB6C5CBED10 and investigation number 1387.

### 2.2. Study Population

The study was conducted in the Hospital Nacional Ramiro Prialé (HNRPP) in the province of Huancayo, Junín—Perú at 3200 masl. A total of 64 patients were enrolled between August 2020 and February 2021. To be enrolled, the participants had to have mild/moderate COVID-19, which was defined by the following criteria: less than 10 days of the disease, the fraction of inspired oxygen (FiO_2_) ≤ 32%, and an arterial oxygen pressure (PaO_2_) ≥ 60 mmHg or PaO_2_/FiO_2_ ≥ 250. These criteria were established by an expert consensus at Hospital Nacional Ramiro Prialé, based on oxygenation parameters at high altitudes [21]. Pregnant women were excluded.

The sample size was calculated according to previous reports, with a probability of severe disease for COVID-19 of 70% in hospitalized patients [22], a sample size of 51 patients was obtained. We considered a 20% loss of participants, for which, we finally enrolled 64 patients in the study.

### 2.3. Definition and Clinical Results

Severe COVID-19 was the primary outcome, which was defined by PaO_2_/FiO_2_ < 250, FiO_2_ ≥ 40%, admission to the intensive care unit (ICU), and/or requiring invasive mechanical ventilation.

Clinical, demographic, and laboratory data were obtained from the participants. Laboratory tests at enrollment and during follow-up included: MPV (fL), lactate dehydrogenase LDH (IU/L), hemoglobin (g/dL), WBC count (10^9^/L), lymphocytes (10^9^/L), platelets (10^9^/L), PaO_2_, FiO_2_, oxygen saturation, creatinine (mg/dL), bilirubin (mg/dL), glutamic-oxaloacetic transaminase GOT (U/L), glutamic pyruvic transaminase GPT (U/L), alkaline phosphatase AP (UI/L) and prothrombin time PT (seconds). A chest computed tomography was obtained only at enrollment.

Laboratory studies were processed within one hour of obtaining the sample as established in the protocol, and the equipment used for hemocytometry, and biochemistry were Sysmex XN-1000 and Wiener lab CMD800IX1 respectively.

The participants were followed up for 21 days or until the development of the primary outcome (severe disease).

### 2.4. Statistical Analysis

Data were analyzed using Stata 15.0 statistical software (StataCorp, College Station, TX, USA). A data quality review was performed to identify and manage missing values, and outliers, among others.

After having collected the information from the patients, a statistical power calculation was performed with the sample obtained, finding a power of 95% to be able to detect a RR of 2 between the two study groups.

Descriptive analysis was performed according to the nature of the variables, reporting relative or absolute frequencies for categorical variables and measures of central tendency or dispersion for numerical variables.

Bivariate analysis was performed according to the fulfillment of assumptions with the following tests: comparison of proportions X^2^, Fisher’s exact, Mann-Whitney-U, or T-student.

Based on the data collected, a ROC analysis was performed using the Youden index to determine the best cut-off point for MPV to predict COVID-19 severity.

With the established cut-off point, it was decided to categorize the MPV variable and perform a multiple regression analysis. For the multiple regression analysis, a generalized linear model was used to deal with the possibility of non-compliance with the assumptions. A model with Poisson family, log link function and robust variance was used to calculate relative risks, and an adjustment was made for age and gender.

## 3. Results

A total of 64 patients were enrolled at the Hospital Nacional Ramiro Prialé Prialé in the city of Huancayo at 3200 masl. Regarding the descriptive analysis of the population on admission (Table 1), the median age was 48.5 years (IQT 39–64.5), the proportion of women was 51.6%, and the median duration of symptoms prior to enrollment was 7 days. A body mass index greater than normal was noted in 85% of participants, and the proportion of patients with a history of chronic disease was less than 10%.

The most frequent symptoms were chest pain (73%), fever (71%), and dyspnea (67%). In the clinical evaluation, a heart rate of 83pul/min (+/−14.1) and an oxygen saturation of 90% (+/−3.63) were found. The median time to develop severe disease from the onset of symptoms was 11 days (IQT 10.5–13).

Regarding laboratory studies, the median hemoglobin was 16.3g/dL (15.3–17.5), the median white blood cell (WBC) count was 6.73 × 10^9^/L (5.25–8.98), the median lymphocyte count was 1.32 × 10^9^/L (0.82–2.10), the median platelet count was 225 × 10^9^/L (175–271), the mean MPV was 9.63 (±0.84), the median LDH was 477 IU/L (404–566). There was a higher proportion of progressive phase in pulmonary computed tomography (60%).

**Figure 1:**
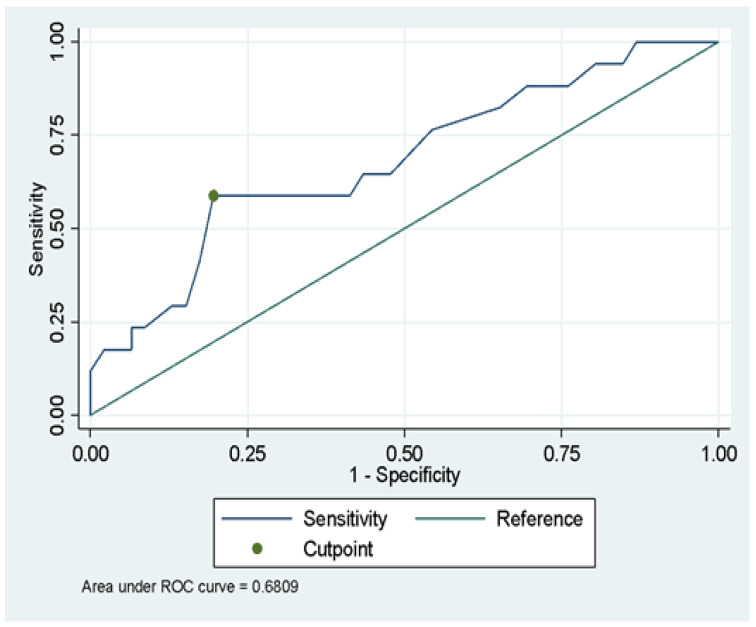
ROC analysis between MPV value and severity of SARS-CoV-2 infection [ROC: 0.6809, 95% CI (0.52664-0.83526)].

For the bivariate analysis, 2 groups were evaluated: (a) Patients who did not progress to severe disease at 21-day follow-up, named as “non-severe” {47 patients (73.4%)}, and (b) Patients who developed severe disease (primary outcome) {17 patients (26.6%)}. Although the study was not designed to evaluate other variables besides MPV, in the bivariate analysis we found that the male gender was associated with a higher proportion of severe disease when compared to the female gender (38.7% vs. 15.2%; *p* < 0.003). A higher median age was also found in patients with the severe disease when compared with non-severe patients (55 vs. 44 years, *p* = 0.04). The mean heart rate was also significantly higher in severe cases (88 vs. 80 lpm, *p* = 0.02), as well as headaches and AST levels (Table 2). When analyzing MPV, we found that the mean MPV was higher in the severe disease group in comparison to the non-severe group (10 vs. 9.5; *p* = 0.023). Thus, a ROC analysis was conducted using the Youden index to choose the best cut-off point of MPV to predict severity (Figure 1 and Table 3). A value of 10.15 fL was established with an AUC of 0.70, Youden index of 0.39, a sensitivity of 59%, a specificity of 80%, a positive predictive value of 53%, and a negative predictive value of 84%. In addition, a positive log-likelihood ratio of 2.95 and a negative log-likelihood ratio of 0.51.

**Figure 2:**
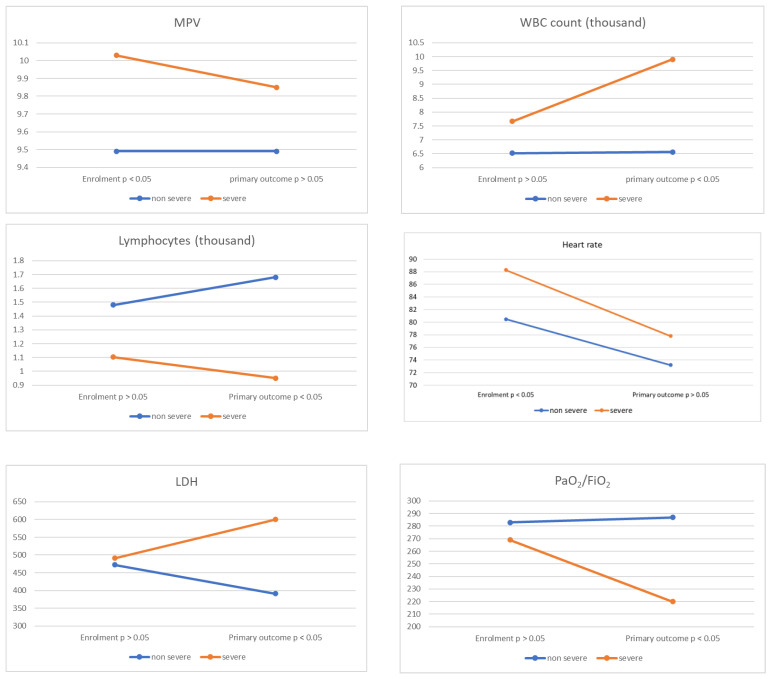
Evolution of laboratory tests from enrolment to primary outcome. (Mann-Whitney-W test, student’s test).

Finally, a regression analysis was conducted (Table 4), finding that patients with MPV equal to or greater than 10.15 fL have a higher risk of presenting severe disease (RR 3.31 95% CI: 1.47–7.43; *p* = 0.004). Upon performing multiple regression analysis adjusted for gender and age, MPV on admission was still a predictor of severe disease (RR 2.93; 95% CI: 1.38–6.23; *p* = 0.005). In addition, the evolution of the patients who developed severe disease was monitored and compared with the rest of the population in the same period, finding an elevation of LDH and WBC count, and a decrease in lymphocytes, MPV, heart rate, and PaO_2_/FiO_2_ in severe cases (Figure 2).

## 4. Discussion

### 4.1. Main Results

In the present study, we found that an elevated MPV in the first week of disease predicted severity in patients with COVID-19 at high altitudes. It is well-known that different laboratory tests are being investigated worldwide to predict COVID-19 and generate interventions to reduce its mortality. However, scientific evidence on the high-altitude population is scarce, therefore, healthcare professionals often use the data obtained at sea level to explain the changes that occur at high altitudes, which could lead to inaccurate conclusions.

### 4.2. Comparison with Previous Studies

MPV has been evaluated in multiple retrospective studies, both alone and in association with other markers. San et al. found that a high MPV/lymphocyte index is associated with severe forms of COVID-19 (*p* < 0.001) [23], this association was also found for a higher level of MPV [24,25,26]. On the other hand, a prognostic model determined that a decreasing trend of MPV can be a useful marker to assess the evolution of the patient with COVID-19 [27]. However, some studies contradict these findings [28,29]. In our study, we found that an MPV > 10.15 fL in the first week of disease can predict severe COVID-19 in people living at high altitudes.

Regarding associated factors, retrospective studies in COVID-19 have found that age, renal, cardiovascular, pulmonary diseases, elevated SOFA score, leukopenia, lymphopenia, elevated LDH, and elevated D-dimer are factors associated with severity and/or mortality in COVID-19. However, these studies have not been conducted in a high-altitude population [3,30,31].

We found that the age, sex, heart rate, headache, MPV, AST, and percentage of lung involvement in pulmonary computed tomography, all of them evaluated during the first week of the disease, were associated with progression to severe forms of COVID-19. WBC count, lymphocytes, LDH, and platelet count during the first week of disease were not associated with progression to severe forms; however, these variables changed in patients who already developed the severe disease (Figure 2), which suggests that these parameters may be more useful for diagnosing severity.

### 4.3. Interpretation of Findings

The WHO uses oxygen saturation as a criterion to define COVID-19 severity, but does not consider values for high altitude populations such as the one living in the city of Huancayo (3200 masl) [21]. In our study, we found a basal mean oxygen saturation of 90.6% for those who did not develop severe disease and 89.5% for those who developed severe disease on follow-up (*p* = 0.32). In addition, a median arterial oxygen pressure of 65.2 mmHg was found in the population studied. These results are different from the ones reported at sea level, such as the study conducted in China, which found a PaO_2_ of 96.64 mmHg for the survivor group and 76.1 mmHg for non-survivors [25]. On the other hand, a study conducted in Quito/Ecuador, a city at 2850 masl, reports a PaO_2_ of 63.3 mmHg for the survivor group and 75.4 mmHg for non-survivors. These divergent results are probably influenced by altitude or by the limitation of being retrospective studies, so their interpretation should be made with caution.

There are also narrative reviews that suggest that people living at high altitudes have a lower risk of becoming infected by this virus due to its low replication which is influenced by hypoxia, humidity, and temperature. Furthermore, there are reports of fewer ACE2 receptors in the target cells of high-altitude populations, therefore a lower probability of virus entry. These data and some descriptive studies suggest that in high-altitude areas, severe forms of COVID are less common [32,33]. Although our study did not aim to analyze the frequency of severity, we found that 26% of our cohort developed severe forms, which is similar to that reported at sea level by the Ministry of Health in Peru [34].

### 4.4. Relevance in Clinical Practice

Knowing that an elevated MPV in the first week of disease can predict severe COVID-19 at high altitude is extremely relevant. Its wide availability and low cost make it an ideal marker to be used in healthcare systems with limited resources. MPV can also help to identify patients at high risk of progression in an early manner to plan adequate treatment strategies.

### 4.5. Limitations and Strengths

The study had some limitations: (1) An adequate sample size to evaluate other risk factors for severity was not available. However, this study provides relevant information to evaluate the main association of interest; (2) Several enrolled patients did not have data for all the evaluated variables, which limited their inclusion in the adjusted regression model; however, the associated factors were selected based on their relevance described in the literature.

Besides MPV, other parameters could predict severity, such as the neutrophil/lymphocyte ratio. In the study conducted by Yildiz et al., a neutrophil/lymphocyte ratio of more than 5.94 increased the mortality risk 3.9 times; however, this study had limitations, because it did not specify the length of disease when the ratio was checked, and they did not distinguish between severe and non-severe cases in the control group [35]. Unfortunately, our study did not evaluate this ratio, we believe that more research should be done to test this parameter, additionally, it is necessary to validate prognosis scores of COVID-19 in altitudes such as NEWS2, COVID-GRAM, and 4C mortality score [36].

## 5. Conclusions

The study has shown that an MPV > 10.15 in the first week of the disease may predict severe COVID-19 independently of age and gender. Therefore, this variable could be helpful to identify patients with a high risk of progression in an early stage of the infection, which could allow the planning of treatment in complicated cases, especially in healthcare centers with limited resources. It is important to conduct future prospective studies with a larger population and at different altitude levels that can provide more evidence about the main association of interest, as well as other risk factors of severity.

## Figures and Tables

**Table 1:** Characteristics of the patients on admission.

Characteristics	n (Missing Values)	n = 64
Demographics		
**Gender**	0	
Female		33 (51.6%)
Male		31 (48.4%)
**Age (years)**	0	48.5 (39–64.5) *
**Duration of disease (days)**	0	7 (6−7) *
**Body mass index**	3	
Normal		9 (14.8%)
Overweight		30 (49.2%)
Obesity		22 (36.1%)
**Comorbidities**		
**Diabetes**	0	2 (3.1%)
**Arterial hypertension**	0	5 (7.8%)
**Chronic pulmonary disease**	0	4 (6.3)
**Signs and symptoms on admission**		
**Heart rate (beats/min)**	0	82.6 (± 14.10)
**Oxygen saturation (%)**	0	90.3 (± 3.63)
**Fever t > 38.3 °C**	1	45 (71%)
**Dyspnea**	1	42 (67%)
**Chest pain**	1	46 (73%)
**Headache**	1	39 (62%)
**Cough**	1	38 (60%)
**Odynophagia**	1	23 (37%)
**Diarrhea**	1	19 (30%)
**Rhinorrhea**	1	24 (38%)
**Admission Laboratory**		
**Hemoglobin (g/dL)**	0	16.25 (15.31–17.45) *
**WBC count (10^9^/L)**	0	6.73 (5.25–8.98) *
**Lymphocytes (10^9^/L)**	0	1.32 (0.82–2.10) *
**Platelets (10^9^/L)**	0	225 (175–271) *
**Mean platelet volume (fL)**	1	9.63 (± 0.84)
**Lactate dehydrogenase (IU/L)**	3	477 (404–566) *
**Creatinine (mg/dl)**	1	0.84 (0.75–1.05) *
**AST (U/L)**	2	43 (29–59.5) *
**ALT (U/L)**	1	60.6 (35–90) *
**Bilirubin T (mg/dL)**	10	0.6 (4–9) *
**Alkaline Phosphatase (IU/L)**	13	231 (190–344) *
**Prothrombin time (sec)**	9	13.76 (± 1.1)
**Arterial oxygen pressure**	4	65.2 (58.45–74) *
**PaO_2_/FiO_2_**	4	276.5 (257–296.5) *
**Images**		
**CT scan (phase)**	**11**	**53**
Progressive		32 (60%)
Consolidative		11 (21%)
Normal		10(19%)
% Compromised		30% (8 Y 40)
% Ground glass		20.64 +/− 16.87

* median and interquartile range.

**Table 2:** Comparison between Severe and Non-severe disease (bivariate analysis).

Variables	Non-Severe n 47 (73.4%)	Severe n 17 (26.6%)	*p* Value	Statistical Test
**Gender**			**0.003**	X^2^ test
Female	28 (84.8%)	5 (15.2%)		
Male	19 (61.3%)	12 (38.7%)		
**Age (years)**	44 (36–65)	55 (47–63)	**0.04**	Mann–Whitney–U test
**Time of disease (days)**	7 (5–7)	7 (6–7)	0.99	Mann–Whitney–U test
**Body mass index**			0.48	Fisher’s exact test
Normal	6 (66.7%)	3 (33.3%)		
Overweight	23 (69.7%)	10 (30.3%)		
Obesity	16 (84.2%)	3 (15.8%)		
**Signs and symptoms on admission**				
**Heart rate (beats/min)**	80.5 (+/−14.2)	88.3 (+/−12.7)	**0.05**	Student’s *t*-test
**Oxygen saturation (%)**	90.6 (+/−3.81)	89.5 (+/−3.04)	0.32	Student’s *t*-test
**Fever t > 38.3 °C**	32 (71.1%)	13 (28.9%)	0.78	Fisher’s exact test
**Dyspnea**	30 (71.4%)	12 (28.8%)	0.68	X^2^ test
**Chest pain**	34 (73.9%)	12 (26.1%)	0.76	Fisher’s exact test
**Headache**	32 (82.1%)	7 (18%)	**0.04**	X^2^ test
**Cough**	26 (68.4%)	12 (31.6%)	0.31	X^2^ test
**Odynophagia**	18 (78.3%)	5 (21.7%)	0.48	X^2^ test
**Diarrhea**	14 (73.7%)	5 (26.3%)	0.94	X^2^ test
**Rhinorrhea**	18 (75%)	6 (25%)	0.78	X^2^ test
**Admission Laboratory**				
**Hemoglobin (g/dL)**	16 (14.9–17.3)	16.8 (16.2–17.6)	0.16	Mann–Whitney–U test
**WBC count (10^9^/L)**	6.52 (5.2–9)	7.67 (5.6–8.7)	0.65	Mann–Whitney–U test
**Lymphocytes (10^9^/L)**	1.48 (0.9–2.2)	1.10 (0.8–1.4)	0.07	Mann–Whitney–U test
**Platelets (10^9^/L)**	235 (169–299)	212 (182–228)	0.34	Mann–Whitney–U test
**Mean platelet volume (fL)**	9.5 (+/–0.81)	10 (+/–0.85)	**0.02**	Student’s *t*-test
**Lactate dehydrogenase (IU/L)**	472 (390–561)	491 (423–569)	0.79	Mann–Whitney–U test
**Creatinine (mg/dL)**	0.83 (0.75–1.01)	0.93 (0.8–1.05)	0.39	Mann–Whitney–U test
**AST (U/L)**	41 (27–55)	56 (43–77)	**0.02**	Mann–Whitney–U test
**ALT (U/L)**	58 (31–90)	65 (58–85)	0.33	Mann–Whitney–U test
**Bilirubin T (mg/dL)**	0.6 (0.5–0.9)	0.65 (0.4–0.8)	0.70	Mann–Whitney–U test
**Alkaline Phosphatase (IU/L)**	241 (198–344)	212.5 (185–309)	0.57	Mann–Whitney–U test
**Prothrombin time**	13.69 (±1.50)	14.08 (±0.86)	0.43	Student’s *t*-test
**Arterial oxygen pressure**	67.8 (59.4–77)	60.2 (56.5–65.4)	0.11	Mann–Whitney–U test
**PaO_2_/FiO_2_**	283 (260–304)	269 (252–278)	0.15	Mann–Whitney–U test
**Images**				
**CT scan (phase)**			0.09	Fisher’s exact test
Progressive	21 (65.6%)	11 (34.4%)		
Consolidative	7 (63.6%)	4 (36.4%)		
Normal	10 (100%)	0 (0%)		
% Compromised	22.7 (+/−18.6)	41.9 (+/−17.1)	**0.001**	Student’s *t*-test
% Ground glass	16.6 (+/−15.3)	30.9 (+/−16.9)	**0.004**	Student’s *t*-test

X^2^ test, Fisher’s exact test, Mann-Whitney-U test, Student’s *t*-test.

**Table 3:** Analysis of the calculated cut-off point for MPV as a predictor of severity of SARS-CoV-2 infection.

Variable	AUC (IC95%)	Cut-Off Point	Youden Index	Sensitivity	Specificity	AUC	LR+	LR−	PPV	NPV
**MPV**	0.68(0.53–0.84)	10.15	0.39	0.59	0.80	0.70	2.95	0.51	0.53	0.84

MPV: mean platelet volume. AUC: area under curve. LR+: likelihood ratio positive. PPV: positive predictive value. NPV: negative predictive value LR−: likelihood ratio negative.

**Table 4:** Association between MPV and severity of SARS-CoV-2 infection. (Poisson family generalized linear regression model with log link and robust variance).

Variables	Bivariate Analysis			Multiple Regression Analysis *		
	RR	95% CI	*p*	RR	95% CI	*p*
**Gender**						
Female	Ref.					
Male	2.55	1.01–6.46	0.048	2.7	1.07–6.79	0.035
**Age**						
Age in years	1.02	1.00–1.05	0.024	1.03	1.01–1.06	0.009
**Mean platelet volume**						
<10.15	Ref.					
≥10.15	3.31	1.47–7.43	0.004	2.93	1.38–6.23	0.005

* Adjusted for age and gender. RR: Relative risk. 95% CI: 95% confidence interval. Ref: Reference.

## References

[1] Wu Z., McGoogan J.M. (2020). Characteristics of and Important Lessons from the Coronavirus Disease 2019 (COVID-19) Outbreak in China: Summary of a Report of 72,314 Cases from the Chinese Center for Disease Control and Prevention. JAMA.

[2] Huang C., Wang Y., Li X., Ren L., Zhao J., Hu Y., Zhang L., Fan G., Xu J., Gu X. (2020). Clinical features of patients infected with 2019 novel coronavirus in Wuhan, China. Lancet.

[3] Zhou F., Yu T., Du R., Fan G., Liu Y., Liu Z., Xiang J., Wang Y., Song B., Gu X. (2020). Clinical course and risk factors for mortality of adult inpatients with COVID-19 in Wuhan, China: A retrospective cohort study. Lancet.

[4] Ozder A. (2020). A novel indicator predicts 2019 novel coronavirus infection in subjects with diabetes. Diabetes Res. Clin. Pract..

[5] Paliogiannis P., Zinellu A., Scano V., Mulas G., De Riu G., Pascale R.M., Arru L.B., Carru C., Pirina P., Mangoni A.A. (2020). Laboratory test alterations in patients with COVID-19 and non COVID-19 interstitial pneumonia: A preliminary report. J. Infect. Dev. Ctries..

[6] Yun H., Sun Z., Wu J., Tang A., Hu M., Xiang Z. (2020). Laboratory data analysis of novel coronavirus (COVID-19) screening in 2510 patients. Clin. Chim. Acta.

[7] Vélez J.L. (2018). ¿El volumen medio plaquetario es un predictor de mortalidad en pacientes sépticos?: Revisión de la literatura. Rev. Med. Herediana.

[8] Ittermann T., Feig M.A., Petersmann A., Radke D., Greinacher A., Völzke H., Thiele T. (2019). Mean platelet volume is more important than age for defining reference intervals of platelet counts. PLoS ONE.

[9] Noris P., Melazzini F., Balduini C.L. (2016). New roles for mean platelet volume measurement in the clinical practice?. Platelets.

[10] Gasparyan A.Y., Ayvazyan L., Mikhailidis D.P., Kitas G.D. (2011). Mean platelet volume: A link between thrombosis and inflammation?. Curr. Pharm. Des..

[11] Tajarernmuang P., Phrommintikul A., Limsukon A., Pothirat C., Chittawatanarat K. (2016). The Role of Mean Platelet Volume as a Predictor of Mortality in Critically Ill Patients: A Systematic Review and Meta-Analysis. Crit. Care Res. Pract..

[12] Bergoli L.C.C., Castanho E.S., Gonçalves S.C., Wainstein R.V., Piardi D., Araújo G., Mossmann M., Krepsky A.M., Wainstein M.V. (2014). Mean Platelet Volume as a Predictor of Major Cardiovascular Outcomes and Final Coronary Flow in Patients Undergoing Primary Percutaneous Coronary Intervention. Rev. Bras. Cardiol. Invasiva.

[13] Zampieri F.G., Ranzani O.T., Sabatoski V., de Souza H.P., Barbeiro H., da Neto L.M.C., Park M., da Silva F.P. (2014). An increase in mean platelet volume after admission is associated with higher mortality in critically ill patients. Ann. Intensiv. Care.

[14] Bommenahalli Gowda S., Gosavi S., Ananda Rao A., Shastry S., Raj S.C., Menon S., Suresh A., Sharma A. (2021). Prognosis of COVID-19: Red Cell Distribution Width, Platelet Distribution Width, and C-Reactive Protein. Cureus.

[15] Accinelli R.A., Leon-Abarca J.A. (2020). At High Altitude COVID-19 Is Less Frequent: The Experience of Peru. Arch. Bronconeumol..

[16] Zubieta-Calleja G., Merino-Luna A., Zubieta-DeUrioste N., Armijo-Subieta N.F., Soliz J., Arias-Reyes C., Escalante-Kanashiro R., Carmona-Suazo J.A., López-Bascope A., Calle-Aracena J.M. (2021). Re: “Mortality Attributed to COVID-19 in High-Altitude Populations” by Woolcott and Bergman. High Alt. Med. Biol..

[17] Ballaz S.J., Pulgar-Sánchez M., Chamorro K., Fernández-Moreira E., Ramírez H., Mora F.X., Fors M. (2021). Common laboratory tests as indicators of COVID-19 severity on admission at high altitude: A single-center retrospective study in Quito (ECUADOR). Clin. Chem. Lab. Med..

[18] Woolcott O.O., Bergman R.N. (2020). Mortality Attributed to COVID-19 in High-Altitude Populations. High Alt. Med. Biol..

[19] Yue H., Bai X., Wang J., Yu Q., Liu W., Pu J., Wang X., Hu J., Xu D., Li X. (2020). Clinical characteristics of coronavirus disease 2019 in Gansu province, China. Ann. Palliat. Med..

[20] Prokop M., Van Everdingen W., van Rees Vellinga T., Quarles van Ufford H., Stöger L., Beenen L., Geurts B., Gietema H., Krdzalic J., Schaefer-Prokop C. (2020). CO-RADS–A categorical CT assessment scheme for patients with suspected COVID-19: Definition and evaluation. Radiology.

[21] COVID-19 Clinical Management: Living Guidance. https://www.who.int/publications-detail-redirect/WHO-2019-nCoV-clinical-2021-1.

[22] Xiong S., Liu L., Lin F., Shi J., Han L., Liu H., He L., Jiang Q., Wang Z., Fu W. (2020). Clinical characteristics of 116 hospitalized patients with COVID-19 in Wuhan, China: A single-centered, retrospective, observational study. BMC Infect. Dis..

[23] Şan İ., Gemcioğlu E., Davutoğlu M., Çatalbaş R., Karabuğa B., Kaptan E., Erden A., Küçükşahin O., Ateş İ., Karaahmetoğlu S. (2021). Which hematological markers have predictive value as early indicators of severe COVID-19 cases in the emergency department?. Turk. J. Med. Sci..

[24] Lanini S., Montaldo C., Nicastri E., Vairo F., Agrati C., Petrosillo N., Scognamiglio P., Antinori A., Puro V., Di Caro A. (2020). COVID-19 disease-Temporal analyses of complete blood count parameters over course of illness, and relationship to patient demographics and management outcomes in survivors and non-survivors: A longitudinal descriptive cohort study. PLoS ONE.

[25] Ouyang S.M., Zhu H.Q., Xie Y.N., Zou Z.S., Zuo H.M., Rao Y.W., Liu X.Y., Zhong B., Chen X. (2020). Temporal changes in laboratory markers of survivors and non-survivors of adult inpatients with COVID-19. BMC Infect. Dis..

[26] Wang H., Xing Y., Yao X., Li Y., Huang J., Tang J., Zhu S., Zhang Y., Xiao J. (2020). Retrospective Study of Clinical Features of COVID-19 in Inpatients and Their Association with Disease Severity. Med. Sci. Monit. Int. Med. J. Exp. Clin. Res..

[27] Tian S., Zhu X., Sun X., Wang J., Zhou Q., Wang C., Chen L., Li S., Xu J. (2020). A Prognostic Model to Predict Recovery of COVID-19 Patients Based on Longitudinal Laboratory Findings. Virol. Sin..

[28] Lin S., Mao W., Zou Q., Lu S., Zheng S. (2021). Associations between hematological parameters and disease severity in patients with SARS-CoV-2 infection. J. Clin. Lab. Anal..

[29] Mertoglu C., Huyut M.T., Arslan Y., Ceylan Y., Coban T.A. (2021). How do routine laboratory tests change in coronavirus disease 2019?. Scand. J. Clin. Lab. Investig..

[30] Han Y., Zhang H., Mu S., Wei W., Jin C., Tong C., Song Z., Zha Y., Xue Y., Gu G. (2020). Lactate dehydrogenase, an independent risk factor of severe COVID-19 patients: A retrospective and observational study. Aging.

[31] Xu P.P., Tian R.H., Luo S., Zu Z.Y., Fan B., Wang X.M., Xu K., Wang J.T., Zhu J., Shi J.C. (2020). Risk factors for adverse clinical outcomes with COVID-19 in China: A multicenter, retrospective, observational study. Theranostics.

[32] Joyce K.E., Weaver S.R., Lucas S.J.E. (2020). Geographic components of SARS-CoV-2 expansion: A hypothesis. J. Appl. Physiol..

[33] Seclén S.N., Nunez-Robles E., Yovera-Aldana M., Arias-Chumpitaz A. (2020). Incidence of COVID-19 infection and prevalence of diabetes, obesity, and hypertension according to altitude in Peruvian population. Diabetes Res. Clin. Pract..

[34] COVID-19 en el Perú-Ministerio del Salud. https://covid19.minsa.gob.pe/sala_situacional.asp.

[35] Yildiz H., Castanares-Zapatero D., Pierman G., Pothen L., De Greef J., Nana F.A. (2021). Validation of Neutrophil-to-Lymphocyte Ratio Cut-off Value Associated with High In-Hospital Mortality in COVID-19 Patients. Int. J. Gen. Med..

[36] Knight S.R., Ho A., Pius R., Buchan I., Carson G., Drake T.M., Dunning J., Fairfield C.J., Gamble C., Green C.A. (2020). Risk stratification of patients admitted to hospital with covid-19 using the ISARIC WHO Clinical Characterization Protocol: Development and validation of the 4C Mortality Score. BMJ.

